# Supplementary material for: Transcriptome Analysis Reveals Vimentin-Induced Disruption of Cell–Cell Associations Augments Breast Cancer Cell Migration
Source: Cells. 2022 Dec 13;11(24):4035. doi: 10.3390/cells11244035 (PMC9776984; doi:10.3390/cells11244035)
Supplement: Supplementary file 1 [file cells-11-04035-s001.zip › DEGs and enrichment analysis.zip!/DEGs and enrichment analysis/WT_VIMvsVector KEGG/GSEA/WT_VIM/EPSTEIN_BARR_VIRUS_INFECTION/EPSTEIN_BARR_VIRUS_INFECTION(HSA05169).html]

Details for gene set EPSTEIN\_BARR\_VIRUS\_INFECTION(HSA05169)[GSEA]

|  || Dataset | fpkm.sample |
| Phenotype | sample.cls |
| Upregulated in class | WT\_VIM |
| GeneSet | EPSTEIN\_BARR\_VIRUS\_INFECTION(HSA05169) |
| Enrichment Score (ES) | 0.83529913 |
| Normalized Enrichment Score (NES) | 1.3989558 |
| Nominal p-value | 0.0 |
| FDR q-value | 0.16499993 |
| FWER p-Value | 0.165 |
Table: GSEA Results Summary

  

Fig 1: Enrichment plot: EPSTEIN\_BARR\_VIRUS\_INFECTION(HSA05169)      
 Profile of the Running ES Score & Positions of GeneSet Members on the Rank Ordered List

  

| PROBE | DESCRIPTION (from dataset) | GENE SYMBOL | GENE\_TITLE | RANK IN GENE LIST | RANK METRIC SCORE | RUNNING ES | CORE ENRICHMENT || 1 | ENSG00000278678 | CYCSP41 |  |  | 404 | Infinity | -0.0069 | Yes |
| 2 | ENSG00000224650 | IGHV3-74 |  |  | 731 | Infinity | -0.0124 | Yes |
| 3 | ENSG00000238061 | AL356273.2 |  |  | 1017 | Infinity | -0.0173 | Yes |
| 4 | ENSG00000276556 | AF254982.1 |  |  | 1030 | Infinity | -0.0175 | Yes |
| 5 | ENSG00000227924 | RBPJP6 |  |  | 1054 | Infinity | -0.0179 | Yes |
| 6 | ENSG00000234937 | AL139128.1 |  |  | 1128 | Infinity | -0.0191 | Yes |
| 7 | ENSG00000283566 | AC243725.1 |  |  | 1481 | Infinity | -0.0251 | Yes |
| 8 | ENSG00000226393 | IFNA20P |  |  | 1728 | Infinity | -0.0293 | Yes |
| 9 | ENSG00000266707 | PSMD7P1 |  |  | 2084 | Infinity | -0.0353 | Yes |
| 10 | ENSG00000267430 | AC036176.2 |  |  | 2163 | Infinity | -0.0367 | Yes |
| 11 | novel.621 | - |  |  | 2179 | Infinity | -0.0369 | Yes |
| 12 | ENSG00000262870 | CYCSP40 |  |  | 2298 | Infinity | -0.0389 | Yes |
| 13 | ENSG00000275929 | AC068831.5 |  |  | 2380 | Infinity | -0.0403 | Yes |
| 14 | ENSG00000026025 | VIM |  |  | 2388 | 1709.539 | 0.8036 | Yes |
| 15 | novel.668 | - |  |  | 2403 | 30.341 | 0.8183 | Yes |
| 16 | ENSG00000020633 | RUNX3 |  |  | 2457 | 14.705 | 0.8247 | Yes |
| 17 | novel.507 | - |  |  | 2498 | 11.391 | 0.8296 | Yes |
| 18 | ENSG00000184844 | CYCSP45 |  |  | 2576 | 8.609 | 0.8325 | Yes |
| 19 | ENSG00000206503 | HLA-A |  |  | 2636 | 7.641 | 0.8353 | Yes |
| 20 | novel.360 | - |  |  | 3223 | 4.178 | 0.8274 | No |
| 21 | ENSG00000171791 | BCL2 |  |  | 3339 | 3.945 | 0.8274 | No |
| 22 | ENSG00000013275 | PSMC4 |  |  | 3347 | 3.905 | 0.8292 | No |
| 23 | ENSG00000271711 | AC069236.1 |  |  | 3361 | 3.853 | 0.8308 | No |
| 24 | ENSG00000101412 | E2F1 |  |  | 3381 | 3.812 | 0.8324 | No |
| 25 | ENSG00000145386 | CCNA2 |  |  | 3873 | 3.179 | 0.8256 | No |
| 26 | ENSG00000214429 | CYCSP6 |  |  | 4168 | 2.884 | 0.8220 | No |
| 27 | ENSG00000026508 | CD44 |  |  | 4248 | 2.820 | 0.8221 | No |
| 28 | ENSG00000227742 | CALR4P |  |  | 4347 | 2.771 | 0.8218 | No |
| 29 | novel.622 | - |  |  | 4417 | 2.713 | 0.8219 | No |
| 30 | novel.277 | - |  |  | 4462 | 2.674 | 0.8225 | No |
| 31 | novel.605 | - |  |  | 4662 | 2.579 | 0.8204 | No |
| 32 | ENSG00000145604 | SKP2 |  |  | 4725 | 2.541 | 0.8206 | No |
| 33 | ENSG00000135446 | CDK4 |  |  | 4816 | 2.481 | 0.8203 | No |
| 34 | ENSG00000142208 | AKT1 |  |  | 4955 | 2.431 | 0.8191 | No |
| 35 | ENSG00000228818 | AL359918.1 |  |  | 5018 | 2.394 | 0.8192 | No |
| 36 | ENSG00000164105 | SAP30 |  |  | 5103 | 2.346 | 0.8190 | No |
| 37 | novel.603 | - |  |  | 5187 | 2.314 | 0.8187 | No |
| 38 | ENSG00000165916 | PSMC3 |  |  | 5498 | 2.199 | 0.8145 | No |
| 39 | ENSG00000159352 | PSMD4 |  |  | 5532 | 2.188 | 0.8150 | No |
| 40 | ENSG00000184216 | IRAK1 |  |  | 5881 | 2.069 | 0.8101 | No |
| 41 | ENSG00000099860 | GADD45B |  |  | 6115 | 1.999 | 0.8071 | No |
| 42 | ENSG00000179218 | CALR |  |  | 6278 | 1.979 | 0.8053 | No |
| 43 | ENSG00000108344 | PSMD3 |  |  | 6385 | 1.951 | 0.8045 | No |
| 44 | ENSG00000100324 | TAB1 |  |  | 6406 | 1.945 | 0.8051 | No |
| 45 | ENSG00000130706 | ADRM1 |  |  | 6477 | 1.930 | 0.8049 | No |
| 46 | ENSG00000156711 | MAPK13 |  |  | 6748 | 1.866 | 0.8012 | No |
| 47 | ENSG00000224677 | PDIA3P2 |  |  | 6893 | 1.843 | 0.7996 | No |
| 48 | ENSG00000240854 | AC117409.1 |  |  | 6894 | 1.842 | 0.8006 | No |
| 49 | ENSG00000136997 | MYC |  |  | 7014 | 1.815 | 0.7994 | No |
| 50 | ENSG00000198502 | HLA-DRB5 |  |  | 7068 | 1.802 | 0.7994 | No |
| 51 | ENSG00000099341 | PSMD8 |  |  | 7097 | 1.794 | 0.7998 | No |
| 52 | ENSG00000123374 | CDK2 |  |  | 7276 | 1.759 | 0.7976 | No |
| 53 | ENSG00000112576 | CCND3 |  |  | 7396 | 1.736 | 0.7965 | No |
| 54 | ENSG00000105639 | JAK3 |  |  | 7747 | 1.667 | 0.7913 | No |
| 55 | ENSG00000175166 | PSMD2 |  |  | 7841 | 1.650 | 0.7906 | No |
| 56 | ENSG00000087191 | PSMC5 |  |  | 7915 | 1.638 | 0.7901 | No |
| 57 | ENSG00000196498 | NCOR2 |  |  | 7919 | 1.638 | 0.7909 | No |
| 58 | ENSG00000275882 | IKBKGP1 |  |  | 8025 | 1.619 | 0.7899 | No |
| 59 | ENSG00000145675 | PIK3R1 |  |  | 8151 | 1.601 | 0.7886 | No |
| 60 | ENSG00000134574 | DDB2 |  |  | 8161 | 1.599 | 0.7892 | No |
| 61 | ENSG00000015475 | BID |  |  | 8203 | 1.593 | 0.7893 | No |
| 62 | ENSG00000126456 | IRF3 |  |  | 8256 | 1.584 | 0.7892 | No |
| 63 | ENSG00000169375 | SIN3A |  |  | 8302 | 1.578 | 0.7892 | No |
| 64 | ENSG00000175305 | CCNE2 |  |  | 8401 | 1.561 | 0.7883 | No |
| 65 | ENSG00000188833 | ENTPD8 |  |  | 8497 | 1.547 | 0.7874 | No |
| 66 | ENSG00000105221 | AKT2 |  |  | 8603 | 1.535 | 0.7864 | No |
| 67 | ENSG00000105397 | TYK2 |  |  | 8657 | 1.527 | 0.7862 | No |
| 68 | ENSG00000127191 | TRAF2 |  |  | 8817 | 1.506 | 0.7843 | No |
| 69 | ENSG00000255993 | PSMC1P9 |  |  | 8858 | 1.499 | 0.7843 | No |
| 70 | ENSG00000204188 | GGNBP1 |  |  | 8912 | 1.494 | 0.7842 | No |
| 71 | ENSG00000130222 | GADD45G |  |  | 9330 | 1.439 | 0.7778 | No |
| 72 | ENSG00000141510 | TP53 |  |  | 9380 | 1.433 | 0.7776 | No |
| 73 | novel.614 | - |  |  | 9404 | 1.430 | 0.7780 | No |
| 74 | ENSG00000185627 | PSMD13 |  |  | 9433 | 1.425 | 0.7782 | No |
| 75 | ENSG00000111276 | CDKN1B |  |  | 9723 | 1.392 | 0.7739 | No |
| 76 | ENSG00000087088 | BAX |  |  | 9776 | 1.386 | 0.7737 | No |
| 77 | ENSG00000187608 | ISG15 |  |  | 9908 | 1.370 | 0.7722 | No |
| 78 | ENSG00000076984 | MAP2K7 |  |  | 10376 | 1.318 | 0.7649 | No |
| 79 | novel.156 | - |  |  | 10427 | 1.318 | 0.7647 | No |
| 80 | ENSG00000229391 | HLA-DRB6 |  |  | 10539 | 1.312 | 0.7634 | No |
| 81 | ENSG00000103035 | PSMD7 |  |  | 10562 | 1.310 | 0.7637 | No |
| 82 | ENSG00000162888 | C1orf147 |  |  | 10704 | 1.294 | 0.7619 | No |
| 83 | novel.604 | - |  |  | 10733 | 1.291 | 0.7621 | No |
| 84 | ENSG00000030110 | BAK1 |  |  | 10737 | 1.290 | 0.7627 | No |
| 85 | ENSG00000249936 | RAC1P2 |  |  | 10812 | 1.283 | 0.7621 | No |
| 86 | ENSG00000006062 | MAP3K14 |  |  | 10863 | 1.278 | 0.7618 | No |
| 87 | ENSG00000241506 | PSMC1P1 |  |  | 10937 | 1.272 | 0.7612 | No |
| 88 | ENSG00000185386 | MAPK11 |  |  | 10944 | 1.271 | 0.7617 | No |
| 89 | ENSG00000171608 | PIK3CD |  |  | 11010 | 1.264 | 0.7613 | No |
| 90 | ENSG00000226332 | AL354836.1 |  |  | 11025 | 1.262 | 0.7616 | No |
| 91 | ENSG00000171855 | IFNB1 |  |  | 11305 | 1.236 | 0.7575 | No |
| 92 | ENSG00000175730 | BAK1P1 |  |  | 11604 | 1.219 | 0.7530 | No |
| 93 | ENSG00000146232 | NFKBIE |  |  | 11708 | 1.209 | 0.7519 | No |
| 94 | ENSG00000235859 | AC006978.1 |  |  | 11927 | 1.188 | 0.7487 | No |
| 95 | ENSG00000176268 | CYCSP34 |  |  | 11986 | 1.182 | 0.7483 | No |
| 96 | ENSG00000269335 | IKBKG |  |  | 12127 | 1.171 | 0.7465 | No |
| 97 | ENSG00000111331 | OAS3 |  |  | 12135 | 1.170 | 0.7470 | No |
| 98 | ENSG00000117322 | CR2 |  |  | 12267 | 1.158 | 0.7453 | No |
| 99 | ENSG00000034152 | MAP2K3 |  |  | 12350 | 1.153 | 0.7445 | No |
| 100 | ENSG00000263528 | IKBKE |  |  | 12496 | 1.141 | 0.7426 | No |
| 101 | ENSG00000133101 | CCNA1 |  |  | 12585 | 1.133 | 0.7416 | No |
| 102 | ENSG00000116478 | HDAC1 |  |  | 12725 | 1.122 | 0.7398 | No |
| 103 | ENSG00000197943 | PLCG2 |  |  | 12777 | 1.118 | 0.7395 | No |
| 104 | ENSG00000115233 | PSMD14 |  |  | 12846 | 1.113 | 0.7389 | No |
| 105 | novel.643 | - |  |  | 12866 | 1.111 | 0.7391 | No |
| 106 | ENSG00000100906 | NFKBIA |  |  | 12989 | 1.103 | 0.7376 | No |
| 107 | ENSG00000232901 | CYCSP10 |  |  | 13067 | 1.097 | 0.7368 | No |
| 108 | ENSG00000127922 | SEM1 |  |  | 13140 | 1.091 | 0.7361 | No |
| 109 | ENSG00000153094 | BCL2L11 |  |  | 13248 | 1.083 | 0.7348 | No |
| 110 | ENSG00000108984 | MAP2K6 |  |  | 13252 | 1.083 | 0.7353 | No |
| 111 | ENSG00000104825 | NFKBIB |  |  | 13328 | 1.077 | 0.7346 | No |
| 112 | ENSG00000108671 | PSMD11 |  |  | 13366 | 1.075 | 0.7345 | No |
| 113 | ENSG00000110092 | CCND1 |  |  | 13381 | 1.074 | 0.7348 | No |
| 114 | ENSG00000254087 | LYN |  |  | 13520 | 1.062 | 0.7329 | No |
| 115 | ENSG00000168040 | FADD |  |  | 13529 | 1.061 | 0.7333 | No |
| 116 | ENSG00000173039 | RELA |  |  | 13620 | 1.055 | 0.7323 | No |
| 117 | ENSG00000232027 | AL671986.1 |  |  | 13700 | 1.049 | 0.7315 | No |
| 118 | ENSG00000167004 | PDIA3 |  |  | 13788 | 1.043 | 0.7305 | No |
| 119 | ENSG00000187555 | USP7 |  |  | 13906 | 1.034 | 0.7290 | No |
| 120 | ENSG00000173692 | PSMD1 |  |  | 13934 | 1.031 | 0.7291 | No |
| 121 | ENSG00000197170 | PSMD12 |  |  | 13975 | 1.028 | 0.7289 | No |
| 122 | ENSG00000102871 | TRADD |  |  | 14037 | 1.023 | 0.7284 | No |
| 123 | ENSG00000161057 | PSMC2 |  |  | 14141 | 1.015 | 0.7271 | No |
| 124 | ENSG00000165025 | SYK |  |  | 14231 | 1.008 | 0.7261 | No |
| 125 | ENSG00000131323 | TRAF3 |  |  | 14300 | 1.003 | 0.7254 | No |
| 126 | ENSG00000163636 | PSMD6 |  |  | 14699 | 0.989 | 0.7191 | No |
| 127 | novel.685 | - |  |  | 15034 | 0.968 | 0.7139 | No |
| 128 | ENSG00000172936 | MYD88 |  |  | 15106 | 0.964 | 0.7132 | No |
| 129 | ENSG00000100764 | PSMC1 |  |  | 15433 | 0.944 | 0.7081 | No |
| 130 | ENSG00000112242 | E2F3 |  |  | 15451 | 0.942 | 0.7083 | No |
| 131 | ENSG00000259547 | CYCSP2 |  |  | 15663 | 0.929 | 0.7051 | No |
| 132 | ENSG00000269904 | MAP2K4P1 |  |  | 15954 | 0.922 | 0.7006 | No |
| 133 | ENSG00000136238 | RAC1 |  |  | 16050 | 0.916 | 0.6995 | No |
| 134 | ENSG00000180867 | PDIA3P1 |  |  | 16062 | 0.915 | 0.6997 | No |
| 135 | ENSG00000172115 | CYCS |  |  | 16228 | 0.905 | 0.6974 | No |
| 136 | ENSG00000196591 | HDAC2 |  |  | 16306 | 0.899 | 0.6965 | No |
| 137 | ENSG00000139687 | RB1 |  |  | 16638 | 0.880 | 0.6913 | No |
| 138 | ENSG00000050748 | MAPK9 |  |  | 16713 | 0.875 | 0.6905 | No |
| 139 | ENSG00000088888 | MAVS |  |  | 16843 | 0.867 | 0.6887 | No |
| 140 | ENSG00000105173 | CCNE1 |  |  | 17069 | 0.852 | 0.6853 | No |
| 141 | ENSG00000135341 | MAP3K7 |  |  | 17194 | 0.845 | 0.6836 | No |
| 142 | ENSG00000137275 | RIPK1 |  |  | 17283 | 0.839 | 0.6825 | No |
| 143 | ENSG00000164305 | CASP3 |  |  | 17672 | 0.818 | 0.6763 | No |
| 144 | ENSG00000213341 | CHUK |  |  | 17817 | 0.810 | 0.6742 | No |
| 145 | ENSG00000175104 | TRAF6 |  |  | 18271 | 0.783 | 0.6669 | No |
| 146 | novel.665 | - |  |  | 18536 | 0.770 | 0.6628 | No |
| 147 | ENSG00000138185 | ENTPD1 |  |  | 18538 | 0.770 | 0.6631 | No |
| 148 | ENSG00000159110 | IFNAR2 |  |  | 18544 | 0.769 | 0.6634 | No |
| 149 | ENSG00000095585 | BLNK |  |  | 18612 | 0.764 | 0.6627 | No |
| 150 | ENSG00000100603 | SNW1 |  |  | 18682 | 0.761 | 0.6619 | No |
| 151 | ENSG00000055332 | EIF2AK2 |  |  | 18711 | 0.759 | 0.6618 | No |
| 152 | ENSG00000105647 | PIK3R2 |  |  | 18727 | 0.758 | 0.6619 | No |
| 153 | ENSG00000168214 | RBPJ |  |  | 18742 | 0.757 | 0.6620 | No |
| 154 | ENSG00000267278 | MAP3K14-AS1 |  |  | 18752 | 0.756 | 0.6622 | No |
| 155 | ENSG00000104365 | IKBKB |  |  | 18846 | 0.751 | 0.6610 | No |
| 156 | ENSG00000107643 | MAPK8 |  |  | 18889 | 0.749 | 0.6607 | No |
| 157 | ENSG00000112062 | MAPK14 |  |  | 18949 | 0.746 | 0.6601 | No |
| 158 | ENSG00000064012 | CASP8 |  |  | 18979 | 0.744 | 0.6599 | No |
| 159 | ENSG00000124762 | CDKN1A |  |  | 19129 | 0.735 | 0.6577 | No |
| 160 | ENSG00000077150 | NFKB2 |  |  | 19190 | 0.733 | 0.6571 | No |
| 161 | novel.686 | - |  |  | 19249 | 0.729 | 0.6565 | No |
| 162 | ENSG00000109320 | NFKB1 |  |  | 19286 | 0.727 | 0.6562 | No |
| 163 | ENSG00000118971 | CCND2 |  |  | 19488 | 0.716 | 0.6531 | No |
| 164 | ENSG00000138433 | CIR1 |  |  | 19905 | 0.694 | 0.6464 | No |
| 165 | ENSG00000055208 | TAB2 |  |  | 20022 | 0.687 | 0.6447 | No |
| 166 | ENSG00000116717 | GADD45A |  |  | 20075 | 0.685 | 0.6442 | No |
| 167 | ENSG00000179344 | HLA-DQB1 |  |  | 20131 | 0.681 | 0.6436 | No |
| 168 | ENSG00000100519 | PSMC6 |  |  | 20157 | 0.680 | 0.6435 | No |
| 169 | ENSG00000236814 | AC046176.1 |  |  | 20309 | 0.671 | 0.6413 | No |
| 170 | ENSG00000122008 | POLK |  |  | 20328 | 0.669 | 0.6413 | No |
| 171 | ENSG00000115415 | STAT1 |  |  | 20351 | 0.668 | 0.6412 | No |
| 172 | ENSG00000275345 | AC091167.5 |  |  | 20726 | 0.648 | 0.6352 | No |
| 173 | ENSG00000065559 | MAP2K4 |  |  | 20861 | 0.643 | 0.6332 | No |
| 174 | ENSG00000069869 | NEDD4 |  |  | 20864 | 0.642 | 0.6335 | No |
| 175 | ENSG00000213928 | IRF9 |  |  | 20970 | 0.636 | 0.6320 | No |
| 176 | ENSG00000005844 | ITGAL |  |  | 21024 | 0.635 | 0.6314 | No |
| 177 | ENSG00000142166 | IFNAR1 |  |  | 21030 | 0.635 | 0.6317 | No |
| 178 | ENSG00000117461 | PIK3R3 |  |  | 21106 | 0.632 | 0.6307 | No |
| 179 | ENSG00000116815 | CD58 |  |  | 21123 | 0.630 | 0.6307 | No |
| 180 | novel.671 | - |  |  | 21168 | 0.627 | 0.6303 | No |
| 181 | ENSG00000177853 | ZNF518A |  |  | 21248 | 0.623 | 0.6293 | No |
| 182 | ENSG00000121879 | PIK3CA |  |  | 21520 | 0.608 | 0.6249 | No |
| 183 | ENSG00000240135 | PSMD12P |  |  | 21531 | 0.607 | 0.6251 | No |
| 184 | ENSG00000183735 | TBK1 |  |  | 21555 | 0.605 | 0.6250 | No |
| 185 | ENSG00000104856 | RELB |  |  | 21693 | 0.596 | 0.6229 | No |
| 186 | ENSG00000105810 | CDK6 |  |  | 21736 | 0.593 | 0.6225 | No |
| 187 | ENSG00000135679 | MDM2 |  |  | 21791 | 0.590 | 0.6219 | No |
| 188 | novel.340 | - |  |  | 22014 | 0.576 | 0.6184 | No |
| 189 | ENSG00000170581 | STAT2 |  |  | 22184 | 0.565 | 0.6158 | No |
| 190 | ENSG00000177606 | JUN |  |  | 22299 | 0.557 | 0.6141 | No |
| 191 | ENSG00000198001 | IRAK4 |  |  | 22454 | 0.547 | 0.6118 | No |
| 192 | ENSG00000162434 | JAK1 |  |  | 22497 | 0.544 | 0.6113 | No |
| 193 | novel.616 | - |  |  | 22546 | 0.541 | 0.6108 | No |
| 194 | ENSG00000168610 | STAT3 |  |  | 22678 | 0.531 | 0.6088 | No |
| 195 | ENSG00000051382 | PIK3CB |  |  | 22747 | 0.525 | 0.6079 | No |
| 196 | ENSG00000229124 | VIM-AS1 |  |  | 22784 | 0.523 | 0.6075 | No |
| 197 | ENSG00000132906 | CASP9 |  |  | 22789 | 0.523 | 0.6077 | No |
| 198 | ENSG00000090339 | ICAM1 |  |  | 22798 | 0.521 | 0.6078 | No |
| 199 | ENSG00000082512 | TRAF5 |  |  | 22846 | 0.518 | 0.6073 | No |
| 200 | ENSG00000166710 | B2M |  |  | 22893 | 0.515 | 0.6068 | No |
| 201 | ENSG00000259972 | AC009120.2 |  |  | 22976 | 0.508 | 0.6056 | No |
| 202 | ENSG00000114315 | HES1 |  |  | 22995 | 0.506 | 0.6056 | No |
| 203 | ENSG00000109339 | MAPK10 |  |  | 23256 | 0.491 | 0.6014 | No |
| 204 | ENSG00000010671 | BTK |  |  | 23561 | 0.473 | 0.5964 | No |
| 205 | ENSG00000120868 | APAF1 |  |  | 23647 | 0.466 | 0.5952 | No |
| 206 | ENSG00000026103 | FAS |  |  | 23853 | 0.450 | 0.5919 | No |
| 207 | ENSG00000124232 | RBPJL |  |  | 23884 | 0.448 | 0.5916 | No |
| 208 | ENSG00000259728 | LINC00933 |  |  | 23900 | 0.446 | 0.5916 | No |
| 209 | ENSG00000107201 | DDX58 |  |  | 24203 | 0.420 | 0.5867 | No |
| 210 | novel.623 | - |  |  | 24215 | 0.419 | 0.5867 | No |
| 211 | ENSG00000118503 | TNFAIP3 |  |  | 24342 | 0.410 | 0.5847 | No |
| 212 | ENSG00000235700 | CYCSP52 |  |  | 24493 | 0.399 | 0.5824 | No |
| 213 | ENSG00000198821 | CD247 |  |  | 24644 | 0.387 | 0.5800 | No |
| 214 | ENSG00000185507 | IRF7 |  |  | 24723 | 0.381 | 0.5789 | No |
| 215 | ENSG00000196735 | HLA-DQA1 |  |  | 24861 | 0.367 | 0.5767 | No |
| 216 | ENSG00000089127 | OAS1 |  |  | 25017 | 0.351 | 0.5742 | No |
| 217 | ENSG00000204267 | TAP2 |  |  | 25079 | 0.342 | 0.5734 | No |
| 218 | ENSG00000169245 | CXCL10 |  |  | 25291 | 0.323 | 0.5699 | No |
| 219 | ENSG00000224473 | CCND3P1 |  |  | 25376 | 0.317 | 0.5687 | No |
| 220 | ENSG00000227666 | CYCSP24 |  |  | 25470 | 0.310 | 0.5672 | No |
| 221 | ENSG00000104921 | FCER2 |  |  | 25512 | 0.307 | 0.5667 | No |
| 222 | ENSG00000231925 | TAPBP |  |  | 25541 | 0.303 | 0.5664 | No |
| 223 | ENSG00000203435 | E2F3P2 |  |  | 25666 | 0.286 | 0.5644 | No |
| 224 | ENSG00000136244 | IL6 |  |  | 25778 | 0.273 | 0.5626 | No |
| 225 | ENSG00000204642 | HLA-F |  |  | 25977 | 0.249 | 0.5594 | No |
| 226 | ENSG00000243838 | PSMC1P7 |  |  | 26011 | 0.247 | 0.5589 | No |
| 227 | ENSG00000111335 | OAS2 |  |  | 26204 | 0.229 | 0.5558 | No |
| 228 | ENSG00000137462 | TLR2 |  |  | 26308 | 0.215 | 0.5541 | No |
| 229 | ENSG00000168032 | ENTPD3 |  |  | 26683 | 0.165 | 0.5478 | No |
| 230 | ENSG00000117020 | AKT3 |  |  | 26963 | 0.119 | 0.5431 | No |
| 231 | novel.663 | - |  |  | 27167 | 0.067 | 0.5397 | No |
| 232 | ENSG00000257452 | AC004551.1 |  |  | 28520 | 0.000 | 0.5167 | No |
| 233 | ENSG00000236680 | AL356000.1 |  |  | 28602 | 0.000 | 0.5153 | No |
| 234 | ENSG00000224416 | IFNA22P |  |  | 28826 | 0.000 | 0.5115 | No |
| 235 | ENSG00000268173 | AC007192.1 |  |  | 29070 | 0.000 | 0.5073 | No |
| 236 | ENSG00000007968 | E2F2 |  |  | 29891 | 0.000 | 0.4934 | No |
| 237 | ENSG00000198851 | CD3E |  |  | 29904 | 0.000 | 0.4932 | No |
| 238 | ENSG00000231404 | RAC1P3 |  |  | 29936 | 0.000 | 0.4926 | No |
| 239 | ENSG00000214810 | CYCSP55 |  |  | 30035 | 0.000 | 0.4910 | No |
| 240 | ENSG00000228264 | PSMD8P1 |  |  | 30366 | 0.000 | 0.4853 | No |
| 241 | novel.503 | - |  |  | 30397 | 0.000 | 0.4848 | No |
| 242 | ENSG00000253146 | CIR1P1 |  |  | 31148 | NaN | 0.4720 | No |
| 243 | ENSG00000211947 | IGHV3-21 |  |  | 31175 | NaN | 0.4716 | No |
| 244 | ENSG00000231389 | HLA-DPA1 |  |  | 31322 | NaN | 0.4691 | No |
| 245 | ENSG00000230521 | HCG4P7 |  |  | 31364 | NaN | 0.4684 | No |
| 246 | ENSG00000147885 | IFNA16 |  |  | 31397 | NaN | 0.4679 | No |
| 247 | ENSG00000204287 | HLA-DRA |  |  | 31438 | NaN | 0.4672 | No |
| 248 | ENSG00000224372 | HLA-N |  |  | 31634 | NaN | 0.4639 | No |
| 249 | ENSG00000232314 | PSMC1P13 |  |  | 31715 | NaN | 0.4625 | No |
| 250 | ENSG00000231461 | HLA-DPA2 |  |  | 31783 | NaN | 0.4614 | No |
| 251 | ENSG00000227766 | HCG4P5 |  |  | 31825 | NaN | 0.4607 | No |
| 252 | ENSG00000211938 | IGHV3-7 |  |  | 31870 | NaN | 0.4600 | No |
| 253 | ENSG00000256075 | AC008033.1 |  |  | 31893 | NaN | 0.4596 | No |
| 254 | ENSG00000211937 | IGHV2-5 |  |  | 31999 | NaN | 0.4578 | No |
| 255 | ENSG00000168394 | TAP1 |  |  | 32151 | NaN | 0.4552 | No |
| 256 | ENSG00000243753 | HLA-L |  |  | 32260 | NaN | 0.4534 | No |
| 257 | ENSG00000278473 | IGHV3-41 |  |  | 32389 | NaN | 0.4512 | No |
| 258 | ENSG00000188512 | AF279873.1 |  |  | 32431 | NaN | 0.4505 | No |
| 259 | ENSG00000259990 | AC133561.3 |  |  | 32435 | NaN | 0.4505 | No |
| 260 | ENSG00000225036 | CDK4P1 |  |  | 32539 | NaN | 0.4487 | No |
| 261 | ENSG00000204622 | HLA-J |  |  | 32562 | NaN | 0.4484 | No |
| 262 | ENSG00000269383 | AC012493.2 |  |  | 32795 | NaN | 0.4444 | No |
| 263 | ENSG00000271423 | CYCSP1 |  |  | 33118 | NaN | 0.4389 | No |
| 264 | ENSG00000260690 | CYCSP39 |  |  | 33212 | NaN | 0.4373 | No |
| 265 | ENSG00000250273 | PSMC1P5 |  |  | 33268 | NaN | 0.4364 | No |
| 266 | ENSG00000271507 | AC025884.3 |  |  | 33599 | NaN | 0.4308 | No |
| 267 | ENSG00000266520 | RAC1P1 |  |  | 33857 | NaN | 0.4264 | No |
| 268 | ENSG00000226488 | LINC01824 |  |  | 33954 | NaN | 0.4248 | No |
| 269 | ENSG00000227262 | HCG4B |  |  | 34222 | NaN | 0.4202 | No |
| 270 | ENSG00000276520 | CR589904.2 |  |  | 34299 | NaN | 0.4189 | No |
| 271 | ENSG00000261346 | AC116348.3 |  |  | 34441 | NaN | 0.4165 | No |
| 272 | ENSG00000229142 | HCG4P8 |  |  | 34543 | NaN | 0.4148 | No |
| 273 | ENSG00000223931 | AC142381.1 |  |  | 34582 | NaN | 0.4142 | No |
| 274 | ENSG00000282639 | IGHV3-64D |  |  | 34703 | NaN | 0.4121 | No |
| 275 | ENSG00000270322 | AC091167.3 |  |  | 34718 | NaN | 0.4119 | No |
| 276 | ENSG00000234217 | RBPJP7 |  |  | 34792 | NaN | 0.4107 | No |
| 277 | ENSG00000267046 | E2F3P1 |  |  | 35277 | NaN | 0.4024 | No |
| 278 | ENSG00000260104 | AC068338.1 |  |  | 35601 | NaN | 0.3969 | No |
| 279 | ENSG00000236348 | PSMC1P10 |  |  | 35822 | NaN | 0.3932 | No |
| 280 | ENSG00000262384 | AC034268.1 |  |  | 35947 | NaN | 0.3911 | No |
| 281 | ENSG00000258554 | AC025162.1 |  |  | 35975 | NaN | 0.3906 | No |
| 282 | ENSG00000232216 | IGHV3-43 |  |  | 35999 | NaN | 0.3902 | No |
| 283 | ENSG00000233545 | CYCSP33 |  |  | 36045 | NaN | 0.3895 | No |
| 284 | ENSG00000254056 | IGHV3-71 |  |  | 36088 | NaN | 0.3888 | No |
| 285 | ENSG00000231195 | IFNA11P |  |  | 36142 | NaN | 0.3879 | No |
| 286 | ENSG00000223841 | AC006484.1 |  |  | 36351 | NaN | 0.3843 | No |
| 287 | ENSG00000137080 | IFNA21 |  |  | 36484 | NaN | 0.3821 | No |
| 288 | ENSG00000254395 | IGHV4-55 |  |  | 36686 | NaN | 0.3787 | No |
| 289 | ENSG00000265836 | CCND3P2 |  |  | 36782 | NaN | 0.3770 | No |
| 290 | ENSG00000234724 | HDAC1P1 |  |  | 36859 | NaN | 0.3758 | No |
| 291 | ENSG00000227949 | CYCSP46 |  |  | 36999 | NaN | 0.3734 | No |
| 292 | ENSG00000279365 | AP000695.3 |  |  | 37041 | NaN | 0.3727 | No |
| 293 | ENSG00000256674 | AC020549.1 |  |  | 37130 | NaN | 0.3712 | No |
| 294 | ENSG00000230795 | HLA-K |  |  | 37165 | NaN | 0.3706 | No |
| 295 | ENSG00000211950 | IGHV1-24 |  |  | 37410 | NaN | 0.3665 | No |
| 296 | ENSG00000120235 | IFNA6 |  |  | 37751 | NaN | 0.3607 | No |
| 297 | ENSG00000270318 | IGHV3OR16-11 |  |  | 37876 | NaN | 0.3586 | No |
| 298 | ENSG00000229120 | CYCSP4 |  |  | 37946 | NaN | 0.3574 | No |
| 299 | ENSG00000230119 | AC123900.1 |  |  | 38438 | NaN | 0.3490 | No |
| 300 | ENSG00000227573 | AC004986.1 |  |  | 38479 | NaN | 0.3484 | No |
| 301 | ENSG00000236616 | BAK1P2 |  |  | 38612 | NaN | 0.3461 | No |
| 302 | novel.595 | - |  |  | 38647 | NaN | 0.3455 | No |
| 303 | novel.594 | - |  |  | 38648 | NaN | 0.3455 | No |
| 304 | ENSG00000211951 | IGHV2-26 |  |  | 39086 | NaN | 0.3381 | No |
| 305 | ENSG00000211952 | IGHV4-28 |  |  | 39087 | NaN | 0.3381 | No |
| 306 | ENSG00000211955 | IGHV3-33 |  |  | 39088 | NaN | 0.3381 | No |
| 307 | ENSG00000211957 | IGHV3-35 |  |  | 39090 | NaN | 0.3381 | No |
| 308 | ENSG00000211958 | IGHV3-38 |  |  | 39091 | NaN | 0.3381 | No |
| 309 | ENSG00000211959 | IGHV4-39 |  |  | 39092 | NaN | 0.3381 | No |
| 310 | ENSG00000237584 | RAC1P4 |  |  | 39494 | NaN | 0.3313 | No |
| 311 | ENSG00000224518 | AC006989.1 |  |  | 39543 | NaN | 0.3305 | No |
| 312 | ENSG00000250914 | AC139783.2 |  |  | 39642 | NaN | 0.3288 | No |
| 313 | ENSG00000234829 | IFNA17 |  |  | 39656 | NaN | 0.3286 | No |
| 314 | ENSG00000254601 | CYCSP26 |  |  | 40176 | NaN | 0.3197 | No |
| 315 | ENSG00000248781 | PSMC1P4 |  |  | 40197 | NaN | 0.3194 | No |
| 316 | ENSG00000211972 | IGHV3-66 |  |  | 40289 | NaN | 0.3179 | No |
| 317 | ENSG00000204261 | PSMB8-AS1 |  |  | 40556 | NaN | 0.3133 | No |
| 318 | ENSG00000233995 | AP000547.1 |  |  | 40567 | NaN | 0.3132 | No |
| 319 | ENSG00000231475 | IGHV4-31 |  |  | 40595 | NaN | 0.3127 | No |
| 320 | ENSG00000248993 | AL645941.2 |  |  | 40610 | NaN | 0.3125 | No |
| 321 | ENSG00000233549 | CYCSP35 |  |  | 40942 | NaN | 0.3068 | No |
| 322 | ENSG00000233548 | CYCSP44 |  |  | 40943 | NaN | 0.3068 | No |
| 323 | ENSG00000270472 | IGHV3OR16-9 |  |  | 41081 | NaN | 0.3045 | No |
| 324 | ENSG00000224240 | CYCSP49 |  |  | 41275 | NaN | 0.3012 | No |
| 325 | ENSG00000181126 | HLA-V |  |  | 41397 | NaN | 0.2992 | No |
| 326 | ENSG00000242574 | HLA-DMB |  |  | 41446 | NaN | 0.2984 | No |
| 327 | ENSG00000254721 | AP000879.1 |  |  | 41589 | NaN | 0.2959 | No |
| 328 | ENSG00000237398 | HLA-DPA3 |  |  | 41625 | NaN | 0.2953 | No |
| 329 | ENSG00000255563 | AC021006.3 |  |  | 41975 | NaN | 0.2894 | No |
| 330 | ENSG00000206341 | HLA-H |  |  | 42028 | NaN | 0.2885 | No |
| 331 | ENSG00000223648 | IGHV3-64 |  |  | 42070 | NaN | 0.2878 | No |
| 332 | ENSG00000249624 | AP000295.1 |  |  | 42426 | NaN | 0.2818 | No |
| 333 | ENSG00000253495 | CYCSP23 |  |  | 42789 | NaN | 0.2756 | No |
| 334 | ENSG00000261332 | AC116348.2 |  |  | 42834 | NaN | 0.2749 | No |
| 335 | ENSG00000204632 | HLA-G |  |  | 42944 | NaN | 0.2730 | No |
| 336 | ENSG00000234745 | HLA-B |  |  | 43150 | NaN | 0.2695 | No |
| 337 | ENSG00000253458 | IGHVII-15-1 |  |  | 43424 | NaN | 0.2649 | No |
| 338 | ENSG00000271178 | IGHV3OR16-13 |  |  | 43445 | NaN | 0.2645 | No |
| 339 | ENSG00000259680 | AC136428.1 |  |  | 43600 | NaN | 0.2619 | No |
| 340 | ENSG00000277096 | AC098590.2 |  |  | 43654 | NaN | 0.2610 | No |
| 341 | ENSG00000223865 | HLA-DPB1 |  |  | 43666 | NaN | 0.2608 | No |
| 342 | ENSG00000213522 | RAC1P5 |  |  | 43671 | NaN | 0.2608 | No |
| 343 | ENSG00000250264 | AL669918.1 |  |  | 43691 | NaN | 0.2605 | No |
| 344 | ENSG00000213310 | CYCSP19 |  |  | 43722 | NaN | 0.2600 | No |
| 345 | ENSG00000256847 | CCND2P1 |  |  | 44079 | NaN | 0.2539 | No |
| 346 | ENSG00000274347 | AC127381.2 |  |  | 44482 | NaN | 0.2470 | No |
| 347 | ENSG00000226126 | PSMC1P12 |  |  | 44649 | NaN | 0.2442 | No |
| 348 | ENSG00000231610 | PIK3CDP1 |  |  | 44917 | NaN | 0.2397 | No |
| 349 | ENSG00000218766 | AL450338.1 |  |  | 44981 | NaN | 0.2386 | No |
| 350 | ENSG00000282600 | IGHV3-69-1 |  |  | 45189 | NaN | 0.2351 | No |
| 351 | ENSG00000259317 | AC044860.3 |  |  | 45307 | NaN | 0.2331 | No |
| 352 | ENSG00000229693 | AL606530.1 |  |  | 45392 | NaN | 0.2317 | No |
| 353 | ENSG00000223691 | AC068044.1 |  |  | 45579 | NaN | 0.2285 | No |
| 354 | ENSG00000248293 | AC008413.1 |  |  | 45920 | NaN | 0.2227 | No |
| 355 | ENSG00000237033 | CASP3P1 |  |  | 45940 | NaN | 0.2224 | No |
| 356 | ENSG00000236637 | IFNA4 |  |  | 45969 | NaN | 0.2219 | No |
| 357 | ENSG00000211933 | IGHV6-1 |  |  | 46010 | NaN | 0.2212 | No |
| 358 | ENSG00000235290 | HLA-W |  |  | 46196 | NaN | 0.2181 | No |
| 359 | ENSG00000233843 | CYCSP48 |  |  | 46206 | NaN | 0.2179 | No |
| 360 | ENSG00000226030 | HLA-DQB3 |  |  | 46208 | NaN | 0.2179 | No |
| 361 | ENSG00000256896 | PSMC1P8 |  |  | 46262 | NaN | 0.2170 | No |
| 362 | ENSG00000271581 | AL671883.2 |  |  | 46336 | NaN | 0.2158 | No |
| 363 | novel.637 | - |  |  | 46400 | NaN | 0.2147 | No |
| 364 | ENSG00000211978 | IGHV5-78 |  |  | 46505 | NaN | 0.2130 | No |
| 365 | ENSG00000211970 | IGHV4-61 |  |  | 46507 | NaN | 0.2129 | No |
| 366 | ENSG00000211976 | IGHV3-73 |  |  | 46508 | NaN | 0.2129 | No |
| 367 | ENSG00000211974 | IGHV2-70D |  |  | 46509 | NaN | 0.2130 | No |
| 368 | ENSG00000227738 | AC092646.1 |  |  | 46524 | NaN | 0.2127 | No |
| 369 | ENSG00000211967 | IGHV3-53 |  |  | 46749 | NaN | 0.2089 | No |
| 370 | ENSG00000229836 | AL671883.1 |  |  | 46753 | NaN | 0.2089 | No |
| 371 | ENSG00000253482 | IGHVII-26-2 |  |  | 47197 | NaN | 0.2013 | No |
| 372 | ENSG00000225698 | IGHV3-72 |  |  | 47239 | NaN | 0.2006 | No |
| 373 | ENSG00000267478 | AP002414.3 |  |  | 47338 | NaN | 0.1990 | No |
| 374 | ENSG00000237541 | HLA-DQA2 |  |  | 47428 | NaN | 0.1974 | No |
| 375 | ENSG00000197476 | AC034105.1 |  |  | 47954 | NaN | 0.1885 | No |
| 376 | ENSG00000160654 | CD3G |  |  | 47988 | NaN | 0.1879 | No |
| 377 | ENSG00000233816 | IFNA13 |  |  | 48078 | NaN | 0.1864 | No |
| 378 | ENSG00000188379 | IFNA2 |  |  | 48141 | NaN | 0.1854 | No |
| 379 | ENSG00000233012 | HDAC1P2 |  |  | 48183 | NaN | 0.1847 | No |
| 380 | ENSG00000270099 | AL365273.2 |  |  | 48500 | NaN | 0.1793 | No |
| 381 | ENSG00000274576 | IGHV2-70 |  |  | 48508 | NaN | 0.1792 | No |
| 382 | ENSG00000234961 | AL133415.1 |  |  | 48561 | NaN | 0.1783 | No |
| 383 | ENSG00000225864 | HCG4P11 |  |  | 48678 | NaN | 0.1763 | No |
| 384 | ENSG00000213781 | PSMC1P2 |  |  | 48955 | NaN | 0.1716 | No |
| 385 | ENSG00000223625 | CYCSP32 |  |  | 49107 | NaN | 0.1691 | No |
| 386 | ENSG00000211942 | IGHV3-13 |  |  | 49687 | NaN | 0.1592 | No |
| 387 | ENSG00000211941 | IGHV3-11 |  |  | 49688 | NaN | 0.1592 | No |
| 388 | ENSG00000211946 | IGHV3-20 |  |  | 49690 | NaN | 0.1592 | No |
| 389 | ENSG00000211944 | IGHV3-16 |  |  | 49691 | NaN | 0.1592 | No |
| 390 | ENSG00000211949 | IGHV3-23 |  |  | 49692 | NaN | 0.1592 | No |
| 391 | ENSG00000224524 | CYCSP42 |  |  | 49696 | NaN | 0.1592 | No |
| 392 | ENSG00000216708 | CIR1P3 |  |  | 49732 | NaN | 0.1586 | No |
| 393 | ENSG00000228078 | HLA-U |  |  | 50015 | NaN | 0.1538 | No |
| 394 | ENSG00000274181 | RBPJP2 |  |  | 50202 | NaN | 0.1506 | No |
| 395 | novel.596 | - |  |  | 50323 | NaN | 0.1486 | No |
| 396 | ENSG00000220548 | VIM2P |  |  | 50683 | NaN | 0.1424 | No |
| 397 | ENSG00000267371 | AP001017.1 |  |  | 51031 | NaN | 0.1365 | No |
| 398 | ENSG00000204592 | HLA-E |  |  | 51170 | NaN | 0.1342 | No |
| 399 | ENSG00000120242 | IFNA8 |  |  | 51359 | NaN | 0.1310 | No |
| 400 | ENSG00000254358 | CYCSP22 |  |  | 51493 | NaN | 0.1287 | No |
| 401 | ENSG00000255444 | CYCSP27 |  |  | 51555 | NaN | 0.1277 | No |
| 402 | ENSG00000242036 | AC128713.1 |  |  | 51784 | NaN | 0.1238 | No |
| 403 | ENSG00000253957 | IGHV3-22 |  |  | 51989 | NaN | 0.1203 | No |
| 404 | ENSG00000223741 | PSMD4P1 |  |  | 52038 | NaN | 0.1195 | No |
| 405 | ENSG00000234333 | CYCSP53 |  |  | 52335 | NaN | 0.1145 | No |
| 406 | ENSG00000259270 | AC044860.2 |  |  | 52793 | NaN | 0.1067 | No |
| 407 | ENSG00000224557 | HLA-DPB2 |  |  | 53075 | NaN | 0.1019 | No |
| 408 | ENSG00000235679 | AL161651.1 |  |  | 53130 | NaN | 0.1010 | No |
| 409 | ENSG00000261548 | HLA-P |  |  | 53280 | NaN | 0.0985 | No |
| 410 | ENSG00000204525 | HLA-C |  |  | 53323 | NaN | 0.0977 | No |
| 411 | ENSG00000271130 | IGHV3OR16-8 |  |  | 53358 | NaN | 0.0972 | No |
| 412 | ENSG00000259513 | CYCSP38 |  |  | 53457 | NaN | 0.0955 | No |
| 413 | ENSG00000270400 | AL512782.1 |  |  | 53638 | NaN | 0.0924 | No |
| 414 | ENSG00000235400 | AC104837.1 |  |  | 53740 | NaN | 0.0907 | No |
| 415 | ENSG00000259490 | IGHV3OR15-7 |  |  | 53830 | NaN | 0.0892 | No |
| 416 | ENSG00000250771 | AC106865.1 |  |  | 53836 | NaN | 0.0891 | No |
| 417 | ENSG00000147873 | IFNA5 |  |  | 53848 | NaN | 0.0890 | No |
| 418 | ENSG00000278510 | AC007908.1 |  |  | 53899 | NaN | 0.0881 | No |
| 419 | ENSG00000270550 | IGHV3-30 |  |  | 54022 | NaN | 0.0860 | No |
| 420 | ENSG00000255920 | CCND2-AS1 |  |  | 54034 | NaN | 0.0858 | No |
| 421 | ENSG00000231706 | CYCSP43 |  |  | 54052 | NaN | 0.0856 | No |
| 422 | ENSG00000177455 | CD19 |  |  | 54322 | NaN | 0.0810 | No |
| 423 | ENSG00000277836 | AC141272.1 |  |  | 54407 | NaN | 0.0796 | No |
| 424 | ENSG00000214042 | IFNA7 |  |  | 54465 | NaN | 0.0786 | No |
| 425 | ENSG00000240416 | CYCSP20 |  |  | 54471 | NaN | 0.0785 | No |
| 426 | ENSG00000231244 | PSMC1P3 |  |  | 54665 | NaN | 0.0752 | No |
| 427 | ENSG00000253714 | IGHVII-53-1 |  |  | 54703 | NaN | 0.0746 | No |
| 428 | ENSG00000167286 | CD3D |  |  | 54729 | NaN | 0.0742 | No |
| 429 | ENSG00000253883 | IGHV3-19 |  |  | 55017 | NaN | 0.0693 | No |
| 430 | ENSG00000253137 | AC092709.1 |  |  | 55020 | NaN | 0.0693 | No |
| 431 | ENSG00000253132 | IGHV3-62 |  |  | 55021 | NaN | 0.0693 | No |
| 432 | ENSG00000275063 | AC233755.1 |  |  | 55145 | NaN | 0.0672 | No |
| 433 | ENSG00000186803 | IFNA10 |  |  | 55258 | NaN | 0.0653 | No |
| 434 | ENSG00000228083 | IFNA14 |  |  | 55349 | NaN | 0.0637 | No |
| 435 | ENSG00000217385 | PSMC1P11 |  |  | 55354 | NaN | 0.0637 | No |
| 436 | ENSG00000176998 | HCG4 |  |  | 55487 | NaN | 0.0614 | No |
| 437 | ENSG00000250215 | CIR1P2 |  |  | 55530 | NaN | 0.0607 | No |
| 438 | ENSG00000237669 | HCG4P3 |  |  | 55531 | NaN | 0.0607 | No |
| 439 | ENSG00000259842 | IGHV3OR16-16 |  |  | 55539 | NaN | 0.0606 | No |
| 440 | ENSG00000259303 | IGHV2OR16-5 |  |  | 55690 | NaN | 0.0581 | No |
| 441 | ENSG00000255003 | CYCSP28 |  |  | 55827 | NaN | 0.0557 | No |
| 442 | ENSG00000204252 | HLA-DOA |  |  | 55863 | NaN | 0.0552 | No |
| 443 | ENSG00000232239 | RBPJP5 |  |  | 55903 | NaN | 0.0545 | No |
| 444 | ENSG00000229357 | CYCSP51 |  |  | 56097 | NaN | 0.0512 | No |
| 445 | ENSG00000231130 | HLA-T |  |  | 56312 | NaN | 0.0476 | No |
| 446 | ENSG00000249909 | CYCTP |  |  | 56461 | NaN | 0.0451 | No |
| 447 | ENSG00000258205 | AC079598.4 |  |  | 56764 | NaN | 0.0399 | No |
| 448 | ENSG00000241106 | HLA-DOB |  |  | 56780 | NaN | 0.0397 | No |
| 449 | ENSG00000232810 | TNF |  |  | 56842 | NaN | 0.0386 | No |
| 450 | novel.625 | - |  |  | 56982 | NaN | 0.0363 | No |
| 451 | ENSG00000224373 | IGHV4-59 |  |  | 56993 | NaN | 0.0361 | No |
| 452 | ENSG00000211965 | IGHV3-49 |  |  | 57083 | NaN | 0.0346 | No |
| 453 | ENSG00000211964 | IGHV3-48 |  |  | 57084 | NaN | 0.0346 | No |
| 454 | ENSG00000211966 | IGHV5-51 |  |  | 57085 | NaN | 0.0346 | No |
| 455 | ENSG00000224263 | CYCSP12 |  |  | 57113 | NaN | 0.0341 | No |
| 456 | ENSG00000240255 | PSMC1P6 |  |  | 57424 | NaN | 0.0289 | No |
| 457 | ENSG00000229217 | CYCSP11 |  |  | 57587 | NaN | 0.0261 | No |
| 458 | ENSG00000227735 | CYCSP5 |  |  | 57828 | NaN | 0.0220 | No |
| 459 | ENSG00000261834 | IGHV3OR16-15 |  |  | 58052 | NaN | 0.0182 | No |
| 460 | ENSG00000283607 | IGHVII-30-21 |  |  | 58191 | NaN | 0.0159 | No |
| 461 | ENSG00000277856 | AC233755.2 |  |  | 58276 | NaN | 0.0144 | No |
| 462 | ENSG00000255370 | CYCSP25 |  |  | 58569 | NaN | 0.0095 | No |
| 463 | ENSG00000255379 | CYCSP29 |  |  | 58573 | NaN | 0.0094 | No |
| 464 | ENSG00000204257 | HLA-DMA |  |  | 58607 | NaN | 0.0089 | No |
| 465 | ENSG00000248827 | AC024587.1 |  |  | 58717 | NaN | 0.0070 | No |
| 466 | ENSG00000197919 | IFNA1 |  |  | 58755 | NaN | 0.0064 | No |
| 467 | ENSG00000226557 | TRAF6P1 |  |  | 58803 | NaN | 0.0056 | No |
| 468 | ENSG00000101017 | CD40 |  |  | 58851 | NaN | 0.0048 | No |
| 469 | ENSG00000264179 | AP000894.1 |  |  | 58905 | NaN | 0.0039 | No |
| 470 | ENSG00000233732 | IGHV3OR16-10 |  |  | 59090 | NaN | 0.0008 | No |
Table: GSEA details [plain text format]

  

Fig 2: EPSTEIN\_BARR\_VIRUS\_INFECTION(HSA05169)      
 Blue-Pink O' Gram in the Space of the Analyzed GeneSet

  

Fig 3: EPSTEIN\_BARR\_VIRUS\_INFECTION(HSA05169): Random ES distribution      
 Gene set null distribution of ES for **EPSTEIN\_BARR\_VIRUS\_INFECTION(HSA05169)**
